# Science communication in China: a critical component of the global science powerhouse

**DOI:** 10.1093/nsr/nwaa035

**Published:** 2020-04-01

**Authors:** Jane Qiu

**Affiliations:** Writes for NSR from Beijing

## Abstract

China has attached a great significance to bringing science to the public—known as *kepu* (科普, ‘science popularization’) or *kexue chuanbo* (科学传播, ‘science dissemination’)—in recent years, partly in response to its unprecedented push for innovation in science and technology. In 2018, it spent 16 billion yuan (US$2.3 billion) on such endeavours, nearly 80% of which was government funding, according to a survey conducted by the Ministry of Science and Technology. With one science-education venue for every million people, approximately 76 million visits were made to the country's 518 general-science museums and 142 million visits were made to 943 museums dedicated to a specific subject matter, such as the Geological Museum of China.

In a forum chaired by *National Science Review*’s executive editor-in-chief, Mu-ming Poo, scientists, journalists and public-information officers discussed the differences in science communication between China and developed nations, the challenges and opportunities of raising scientific literacy in China, how it has played out in a wide range of controversial topics, from stem-cell research to climate change, and the importance of international collaboration.

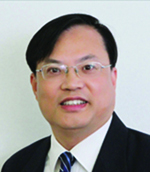

**Tao Deng**

Director of the Institute of Vertebrate Paleontology and Paleoanthropology, Chinese Academy of Sciences, Beijing, China

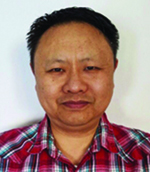

**Hepeng Jia**

Science journalist and science-communication scholar at Soochow University, Suzhou, China

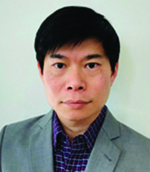

**Brian Lin**

Director of the Editorial Content Strategy, EurekAlert!, American Association of the Advancement of Science, Washington DC, USA

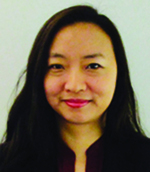

**Joy Ma**

Manager of the Editorial Content, EurekAlert!, American Association of the Advancement of Science, Washington DC, USA

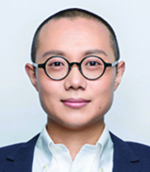

**Lai Xu**

Former chief editor of Guokr.com, Beijing, China

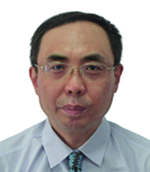

**Shi Yan**

Deputy director of the China Research Institute for Science Popularisation, Chinese Association of Science and Technology, Beijing, China

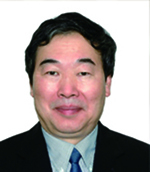

**Mu-ming Poo (Chair)**

Director of the Insitute of Neuroscience, Chinese Academy of Sciences, Shanghai, China

## *KEPU*, *KEXUE CHUANBO* AND SCIENCE COMMUNICATION

**Poo**: What does *kepu* or *kexue chuanbo* mean? Are they the same as science communication that is much talked about in the West?

**Yan**: *Kepu* means science popularization and *kexue chuanbo* means science dissemination. We’ve started to use the term ‘science communication’ in recent years, partly in response to the emergence of science journalism in China and partly to bring us in line with international terminologies. They are similar, interchangeable terms in my view. It's about scientists propagating scientific discovery to the general public as well as public participation in science-related debates. *Kepu* and *kexue chuanbo* probably are more subjective and better suit the situation in China. The key questions are about what we should disseminate and how and through what media.

**Deng**: News reports published in journals such as *Nature* and *Science* are still quite technical and require readers to have some prior insight into the subject in question. By contrast, the *kepu* articles we have in China are much more accessible for people who have little specific knowledge. There are quite a few publications in China that specialize in science popularization. With the exception of *National Science Review*, we don’t have any academic journals that also have a news section.

We are in dire need of good science journalists in China—the middle force between scientists and the public who are able to report science in an accessible and critical way.

—Tao Deng

Regarding science journalism, which is different from *kepu* and *kexue chuanbo*, we are in dire need of good science journalists in China—the middle force between scientists and the public who are able to report science in an accessible and critical way. When there is a major scientific breakthrough, the usual approach in China is for the research institute in question to send a press release to major media outlets. Journalists then write up news reports, often just regurgitating the release.

In the West, accessibility is only a small part of the mix and journalists are a lot more critical and independent in their approach towards reporting scientific developments. They often ask researchers not involved in the study to comment on the possible caveats and significance. I think China lacks this kind of independent science journalism. There are a lot of accessible articles and books about science—but not in-depth critical analyses.

**Poo**: Why is it the case? Is that because of the nature of the Chinese media?

**Deng**: Probably. It may also be because few Chinese science journalists have training in science. They are often trained in journalism, Chinese, literature or foreign languages. They know little of what science is about, let alone special scientific knowledge. They don’t have independent, sound judgement. Their reports are based on press releases provided by scientists or research institutions—without any critical input.

**Jia**: China does have media outlets specialized in science journalism such as *Science and Technology Daily* [published by the Ministry of Science and Technology], *China Science Daily* [published by the Chinese Academy of Sciences], *The Intellectual* and Guokr.com. Many general media groups, such as Xinhua News Agency, *China Daily* and *China News Weekly*, also have dedicated journalists to cover science. So we have a massive force of science journalists, but independent quality science journalism is lacking in China.

This is largely because the function of China's media system is to promote the spirit of scientific discovery and to disseminate scientific knowledge. Chinese journalists are not trained or encouraged to report science from an independent stance. Moreover, few scientists in China have the awareness of their role—and responsibility—in science communication. In the West, scientists are often required to have an outreach component in their projects, especially if they are supported by taxpayers.

## SIMILAR BUT NOT THE SAME

**Poo**: Brian and Joy are the editorial director and editorial manager, respectively, at EurekAlert! [a global news service operated by the American Association for the Advancement of Science (AAAS), the publisher of *Science*] and have regular collaboration with Chinese institutions. What's your view on science communication in China?

**Lin**: The concept of science communication in the West has evolved for decades. It's a widely adopted model. The cornerstone is the neutral stance that journalists adopt when they report scientific practice and progress. The major goal is to provide the public with trustworthy reports. This is deeply rooted in the West but has yet to be fully embraced in China. I think the idea does exist in China, as I’ve seen high-quality independent reports about science by Chinese journalists. In the USA, there is an emphasis on critical and objective appraisals of science—both the good and bad aspects. It's an indispensable aspect of science communication.

**Ma**: There are important differences between science communication and what's known as science popularization/dissemination in China. The former term is used in China increasingly frequently. Many Chinese institutions use science communication to describe what they do—even though the latter terms may be more appropriate. This may cause confusion. It’d be good to make a distinction. It’d be good to provide a clear definition for the two concepts and to clarify their similarities and differences to avoid confusion in practice.

Instead of telling the public what to think, it's about instilling a way of thinking, so they could think for themselves in a critical way and make sound judgement.

—Joy Ma

**Poo**: It's interesting that you don’t think science communication is equivalent to *kepu* or *kexue chanbo.* Why is that?

**Ma**: The aim of science communication is to convey information to the public that is supported by scientific evidence in an accurate and transparent way. It's about arming the public with accurate information and, more crucially, the ability to assess scientific progress and its societal implications in a critical and independent way—rather than just following

the crowds or buying into pseudo science. Science communication goes beyond information dissemination. The ultimate goal is to bring critical thinking to the public. Instead of telling the public what to think, it's about instilling a way of thinking, so they could think for themselves in a critical way and make sound judgement.

By contrast, *kepu* is more about transmitting knowledge rather than a way of thinking. It's about the process of learning about science. It's more preoccupied about transmitting science to the youngsters and to people in rural, poverty-stricken regions than about the exchange of ideas and ways of thinking.

Only when scientific literacy reaches a certain level is the public able to have a proper dialogue with the scientific communities.

—Shi Yan

**Poo**: What is the perspective from the undertaking of Guokr.com?

**Xu**: I think an important reason why science communication in China lacks good journalists is the lack of the market force. In the West, the market is the engine that has produced and cultivated a lot of excellent science journalists and science-communication outlets. Many excellent writers are freelancers. We don’t have this facet of the industry, which is a key constraint of the development of science communication in China.

**Poo**: How do Guokr.com survive financially? What's your business model?

**Xu**: Guokr.com was established in 2010. The goal is to build a bridge between science and the public. I was part of its content department, which has four subdivisions. One team focuses on readers; another team is in close contact with research communities; we also have a video team and a multi-media team. We disseminate frontier scientific progress and respond to readers’ feedback. A third of our content is original; another third are translated articles; the final third are materials from other Chinese websites. Some of the contents target the general public, whereas others target scientific communities, especially young scientists.

We are one of the first media outlets to respond to the rise in social media by setting up specialized Weibo and WeChat teams. All our content go into those platforms—after having adjusted to their specific characteristics. In late 2014, we noted that video products were very popular on the internet and immediately set up a video team. Some of our products are very popular, such as science talk shows, and are on sale on Jingdong and Tmall [which are two of the most popular e-commerce companies in China]. Our e-commerce team has produced species calendar, science books and science-themed videos and toys.

**Poo**: How about official media outlets specialized in science? How do they operate?

**Jia**: Many of them are affiliated with and funded by government administrations. *Science and Technology Daily*, for instance, is affiliated with the Ministry of Science and Technology. *China Science Daily* and *Science News* magazine are affiliated with the Chinese Academy of Sciences. Their main job is to disseminate policy and research development of those administrations. In 2006, Zhao Yan set up a news-aggregating site called ScienceNet.cn. The only original content it produces is blogs. The blogs, which are immensely popular, aim to give voice to scientists at all levels.

## ROLE OF SCIENCE COMMUNICATION IN CONTROVERSIAL TOPICS

**Poo**: What role has science communication played in China in controversial topics such as climate change, genetic modification and stem cells?

**Yan**: This is a very important topic. I think *kepu* (science popularization) and science communication share the same goal, which is to promote citizens’ scientific literacy, especially that of the youngsters. This is crucial if China aspires to be a global leader of science and technology. Without basic scientific literary, there is no point in talking about critical thinking. Since 1992, we have regularly conducted national surveys of scientific literacy. The improvement has been significant, but there are still major gaps compared to developed nations. Only when scientific literacy reaches a certain level is the public able to have a proper dialogue with the scientific communities. That is the crux of the matter.

China has been going through economic transition in the past decades, for which innovation in science and technology is a key driver. Two important questions are at stake here: one is how to cultivate scientists, another is how to prepare the soil for innovation. Here, citizens’ scientific literacy could determine a country's innovation potential—which, in turn, depends on the quality of *kepu* and science communication.

Controversial topics such as climate change, genetic modification and nuclear power have attracted a lot of attention across the country. The level of citizens’ scientific literacy could determine whether those controversies can be resolved in a rational way. It's all part of the mission of *kepu—*which, in my view, has had an important role in communicating and resolving those issues.

Many factors other than scientific literacy have a role in determining the public opinion of science.

—Hepeng Jia

**Jia**: Raising scientific literacy is only part of the solution to controversial issues. The level of scientific literacy in Europe and the USA is a lot higher, but they are not short of scientific controversies. Europe and the USA are also quite different in their citizens’ attitude towards, say, genetically modified (GM) crops and climate change. It's quite complicated. Many factors other than scientific literacy have a role in determining the public opinion of science.

**Yan**: Of course, multiple factors are at play. But basic scientific literacy is absolutely necessary for having a rational public dialogue. If the public doesn’t understand basic biological principles, if they don’t know what DNA is, how could they contribute to a debate about GM crops? In China, some reporters and celebrities are very vocal about certain scientific topics, but you can tell they don’t have a basic understanding of the specific science involved. They have greatly misled the public because they are celebrities and people look up to them.

**Ma**: Government officials are also the target audience for accurate and transparent dissemination of scientific progress, and so science communication can have a significant impact on policy decisions.

**Yan**: I agree. In China's Action Plan of Improving Citizens’ Scientific Literacy—a 15-year plan enacted in 2006 by the State Council—a key target population is indeed civil servants and government officials. This is really important. A lot of policy mistakes are due to the lack of understanding of science and scientific way of thinking.

Scientific literacy of science journalists is also critical. They may not understand what science is about. Yet, they have a lot of control and influence over the outcome of scientific controversies. In China, some media reports suggest GM crops can cause diseases. This has been detrimental to the public perception of this nascent technology in China—which, in turn, has affected policy decisions. A major challenge of communicating complex science to the general public is the need to make it accessible. But there is a fine line between making science accessible and dumbing it down.

**Lin**: Scientific information and scientific literacy are two very different concepts. In *kepu* and science communication, we tend to focus on disseminating scientific information to the general public. But what they do about it once they get the information is quite a different matter. Some people may make a judgement based on whether the information is useful to him personally, like how it could affect housing prices or how it could promote the common good. Our goal should be to help the public to make sound judgement. This is not necessarily related to the quantity of scientific information, but more about appreciating science at a deeper level, what science is about and its process. It's a big challenge all over the world. There is much room for improvement in the USA as well.

## STRONG GOVERNMENT SUPPORT FOR SCIENCE POPULARIZATION

**Poo**: What kind of policies does China have to promote science communication?

**Yan**: China has placed a significant emphasis on *kepu* since the country was established in 1949. The culture ministry had a *kepu* bureau as early as 1958. So *kepu* in China has always been a government-led endeavour. A key policy initiative came about in the late 1990s in the form of Project 2049. Modelled on the Project 2061 spearheaded by the AAAS, it aims to improve science education so that people can become literate in science and technology by 2049, the centenary of the founding of China. It includes a series of research and development initiatives aiming to help educators, communicators, researchers and policymakers to achieve that goal. Key developments are the Law of Science Dissemination, enacted in 2002, and the Action Plan of Improving Citizens’ Scientific Literacy, a 15-year plan promulgated in 2006. Now we are considering the focuses and initiatives for the coming decades.

**Jia**: China should take advantage of its resources and strong infrastructure. I don’t think any other country has so many daily newspapers specialized in science. Each province also has its local science newspapers. This is unique in China and has massive potential. It's worthwhile exploring how this could be combined with the market economy to deliver the maximum impact.

We compete not only with our colleagues, but also with other industries such as computer games, movies and television series.

—Lai Xu

**Poo**: Do we collaborate with institutions in developed nations, such as the AAAS? I know there is Chinese-language version of US publications—such as *huanqiu kexue* (‘Global Science’) for *Scientific American* and *huaxia dili* (‘Chinese Geographic’) for *National Geographic*. This surely has a direct impact on science communication in China.

**Ma**: The AAAS is a massive organization and publishes many scientific journals including *Science* magazine. Both Brian and myself work for EurekAlert, the AAAS’s news-release platform. As part of the science-communication initiative, we’ve had close collaboration with press and information officers at the Chinese Academy of Sciences. A key idea is to introduce to them the most advanced and most practical ideas and approaches in science communication. I feel they have progressed rapidly. They now have a much better understanding of what international media need from them and how their approaches and styles are different from Chinese media. They also have a better idea which media outlets to approach for a particular paper or periodical and how to establish long-term public relations.

**Lin**: There are many departments in the AAAS. As far as China is concerned, a main function of our department is to facilitate communication between journalists and information officers in China, so scientific information can be disseminated accurately and effectively. This kind of operation has a long history in the West, where every university and research institution has a press office to deal with media relations. There are similar infrastructures in China but the ideas are not exactly the same. Our job is to bridge the gap, so communication between journalists and Chinese scientists can be as smooth and effective as possible. This is a key element in science communication.

**Poo**: There should be more initiatives from the government level. In the USA, successful science writers are recruited by universities to teach students how to communicate science to the public. Do we have similar approaches in China?

**Jia**: Some Chinese universities have established majors in science communications. But most of the courses are taught by scholars trained in the philosophy of science rather than science communication. They don’t really have the perspectives of working as information officers or science journalists. Songshuhui-Association of Science Communicators, a grassroots association of science communication in China, has organized training workshops, but the impact is rather limited.

**Poo**: One of the most effective *kepu* initiatives in China is the publication in the 1950s of a book series called *shiwange weishenmo* (‘*A Hundred Thousands Whys*’). It affected a whole generation. Many scientists said they got hooked on science because of the series. I’ve noted an updated edition is still on sale.

**Yan**: I know the project quite well. It was inspired by a similar book published in the former Soviet Union that targeted questions youngsters were most curious about. The initiative started with a collection of questions and then searched for suitable writers. One of the writers, Yonglie Ye, was a student at Peking University at the time, and subsequently became a highly successful professional *kepu* writer. The latest edition has incorporated new questions and the latest scientific progress, and includes an online version.

**Poo**: This is a great success story. Has the Chinese government organized similar writing projects in recent years?

**Yan**: In terms of official initiatives, the Chinese Association of Science and Technology has led several projects, including dozens of book series. One book series was written by academicians [Fellows of Chinese Academy of Sciences and Chinese Academy of Engineering]. But such top-down initiatives have had limited success and might not be the best way to go about it. Most popular-science books in the West are not sponsored through official channels. They are products of the market. I think this is what we need to think more about—that is, how to combine the top-down approach with the market economy to achieve the desired outcome.

**Poo**: How about *kepu* initiatives on the internet?

**Xu**: The Chinese government has had projects on so-called ‘*kepu* information engineering’. Over two dozen institutions, including Guokr.com, participated in the projects and targeted the progress of major scientific programmes such as GM crops. I don’t think we can replicate the success of *A Hundred Thousands Whys*. There were very few cultural products in 1950s, so it was relatively easy to get the attention. Now things are different. We compete not only with our colleagues, but also with other industries such as computer games, movies and television series. Our audience has too many options. At Guokr.com, we are trying new tricks and new approaches all the time to compete for attention. It's tough.

## BRINGING CHINESE SCIENCE TO THE INTERNATIONAL COMMUNITIES

**Poo**: China has invested heavily in science and technology. Universities and research institutions are keen to introduce their progress to the international communities. What are the main approaches? And how is it going?

**Deng**: A main approach is to publish special features in scientific journals, such as *Nature* and *National Science Review*, so the public get to know what we have accomplished. Another approach is to organize exhibitions. This is probably unique to palaeontology. Our institute has had exhibitions, both in China and abroad, on Chinese dinosaur research. They are immensely popular. Many people are aware of dinosaur research in the USA because American scientists are much better at disseminating their work to the public through films, such as *Jurassic Park*, and other means. This has left them with the impression that all dinosaurs are massive and vicious.

Our exhibition has shown that there is great diversity in dinosaurs, big and small, and some had wings. The public gets a more realistic understanding of what dinosaur kingdoms were like and the fascinating insight into bird evolution. Many other research fields, such as neuroscience and space science, can also use this approach to raise the public awareness of the latest scientific development.

In science communication, we need to think more about what the public may be interested in rather than what we want to tell them.

—Brain Lin

**Poo**: I totally agree. Many scientists, especially biologists, get interested in science because of the dinosaur research they came across when they were a kid. Our institute organizes annual *kepu* summer camps in rural regions, which are mostly taught by graduate students. We design some simple experiments, show the kids a few slides of the brain and test their brain function. Exhibitions are another way of going about it, especially by way of a national tour. Such platforms also provide graduate students with an opportunity to explain their work to the public.

Graduate students in our institute have recently contributed to a book called *The Mystery of the Brain*. It was nice *kepu* training for them. I often tell the students that only 10%–20% of them will become scientists and so they need to think about what else they want to do. And we should provide them with the opportunities to try out different possibilities, including *kepu* and science journalism. We don’t need a lot of scientists. What we really need are a lot of high-level professionals who are scientifically literate.

**Jia**: There are definitely advantages in science dissemination done by research institutions. A disadvantage is that they don’t always know what the public want to know or the best way to go about it. The website of the Chinese Academy of Sciences, for instance, is full of the content about research progress and recently published papers. But they are not written in a way that can attract the attention of the public or science journalists.

Another important issue is how scientists can be actively involved in science communication. At the 9th World Conference of Science Journalists, held in San Francisco in October 2017, a popular programme was having lunch with top scientists. Scientists knew that this was a great opportunity to spread the word about their work. This kinds of events are very rare in China. We need to think more about how best to bring the two communities together.

**Lin**: The websites of many research institutes in the West also place a lot of emphasis on research papers. In science communication, we need to think more about what the public may be interested in rather than what we want to tell them. This is absolutely crucial for journalists and popular-science writers. They always have to be clear who their readers are, what they know, what they don’t know and what they want to know. It's a golden rule in science communication.

## EUREKALERT!: A GLOBAL HUB OF SCIENCE COMMUNICATION

**Poo**: Could you explain in greater detail how EurekAlert! operates?

**Lin**: As you know, the AAAS is the world's largest general-science membership society. An important part of its remit is to communicate science broadly. To achieve that end, the AAAS launched EurekAlert! in 1996. It's a news-release distribution service that gathers the latest breaking scientific research in one easily accessible place and gives journalists access to the latest studies before publication. It now provides news in English, Spanish, French, German, Portuguese, Japanese and Chinese.

There is an urgent need for a concerted effort and long-term plans to build the science-communication capacity.

—Mu-ming Poo

**Ma**: The news releases, which can number over 200 a day, are provided by more than 5000 public-information officers from 2300 universities, academic journals, government agencies and medical centres around the world. Over 14 000 reporters from more than 90 countries have registered for free access to embargoed materials. Each month, we have 780 000 independent visitors and 2 million visits in total. In addition to providing timely information, we have a searchable archive containing over 320 000 news releases published over two decades.

The releases on EurekAlert! provide a peg and the basic information for a potential news story. We’ve noted that many journalists in China use large chunks of our releases—and those provided by research institutions—for their media reports without talking to scientists and without critical appraisal of the work. This is not a responsible way of going about it.

**Jia**: Indeed. The correct way is to talk to not only the scientists involved in a particular study, but also those who are not involved in the work for independent comments, especially on the potential caveats and significance of the findings. A large part of science reporting is about striking a balance between simplifying science to make it accessible and not simplifying it too much to make it inaccurate.

**Poo**: The biggest issue in science journalism is distortion and exaggeration. I do understand that media reports can’t be as detailed or as accurate as scientific papers. But a lot of the misinformation in the media is caused by the tendency of media to sensationalize the message to attract readership. For example, a Chinese team published a few papers on their imaging studies of the human cerebral cortex. Some news reports, including at least one by Xinhua [a state news agency], claimed that the team had completed mapping the human cortex. It's a gross exaggeration and totally unacceptable. Senior officials at the Ministry of Science and Technology were shocked when they saw the news because they were in the process of launching China's Brain Project and a focus of the 15-year initiative was to map the human cerebral cortex.

**Ma**: There is an urgent need for China to assemble a team of professional science communicators, consisting of various components of the chain—including scientists, information officers, science journalists and museum staff. China has invested heavily in research and development but not enough in science communication. Perhaps a proportion of science grants could be earmarked for communication or outreach efforts, as is often the case in the USA. Because American scientists are often required to demonstrate the outcome of their outreach efforts—be it media citations, talks in journalism conferences, exhibitions or short documentaries—they take it really seriously. The message is clear: communicating their work to the public is obligatory; it's one of the criteria against which their performance is measured.

**Poo**: This is a great idea. There is a serious lack of experienced science communicators in China. Our institute has recruited someone who used to be an editor at *huanqiu kexue* (‘Global Science’), the Chinese-language version of *Scientific American*, to take the lead on science communication and public relations. At *National Science Review*, we also plan to include high-quality, in-depth reports of science news—just like *Nature* and *Science* do—but it's quite challenging right now. China really needs to work on this from the government level. There is an urgent need for a concerted effort and long-term plans to build the science-communication capacity.

